# Chemically modulated graphene quantum dot for tuning the photoluminescence as novel sensory probe

**DOI:** 10.1038/srep39448

**Published:** 2016-12-19

**Authors:** Eunhee Hwang, Hee Min Hwang, Yonghun Shin, Yeoheung Yoon, Hanleem Lee, Junghee Yang, Sora Bak, Hyoyoung Lee

**Affiliations:** 1 Centre for Integrated Nanostructure Physics (CINAP), Institute of Basic Science (IBS), 2066 Seoburo, Jangan-gu, Suwon 16419, Republic of Korea; 2 Department of Chemistry, Sungkyunkwan University, 2066 Seoburo, Jangan-gu, Suwon 16419, Republic of Korea; 3 Department of Energy Science, Sungkyunkwan University, 2066 Seoburo, Jangan-gu, Suwon 16419, Republic of Korea

## Abstract

A band gap tuning of environmental-friendly graphene quantum dot (GQD) becomes a keen interest for novel applications such as photoluminescence (PL) sensor. Here, for tuning the band gap of GQD, a hexafluorohydroxypropanyl benzene (HFHPB) group acted as a receptor of a chemical warfare agent was chemically attached on the GQD via the diazonium coupling reaction of HFHPB diazonium salt, providing new HFHPB-GQD material. With a help of the electron withdrawing HFHPB group, the energy band gap of the HFHPB-GQD was widened and its PL decay life time decreased. As designed, after addition of dimethyl methyl phosphonate (DMMP), the PL intensity of HFHPB-GQD sensor sharply increased up to approximately 200% through a hydrogen bond with DMMP. The fast response and short recovery time was proven by quartz crystal microbalance (QCM) analysis. This HFHPB-GQD sensor shows highly sensitive to DMMP in comparison with GQD sensor without HFHPB and graphene. In addition, the HFHPB-GQD sensor showed high selectivity only to the phosphonate functional group among many other analytes and also stable enough for real device applications. Thus, the tuning of the band gap of the photoluminescent GQDs may open up new promising strategies for the molecular detection of target substrates.

The technology of making portable devices has developed a lot in recent years. As the growing hazard of chemical attacks in our society grows, a detection of toxic materials still has been widely studied in sensor technology^1^. This technology has improved the quality of life and has contributed to novel applications such as health monitoring, environmental monitoring, and safety. Chemical sensing is of crucial importance because of the growing hazard of terrorist chemical attacks in our society. To protect against the threat of toxic chemicals, easily conformable, cost effective, portable and nontoxic sensory materials are urgently needed in military and civilian systems.

Organophosphates are a well-known example of such chemical warfare agents (CWA) that are highly toxic, volatile and can irreversibly alter enzymes in neurons^2^. It can suppress the activity of acetyl cholinesterase (AChE) and produce the toxic acetylcholine (ACh) which will cause loss of functions on muscle control, breathing or even death. To handle the threat of CWAs, many researchers have developed detectable materials with applicable simulants of CWA. Most of researchers have focused on receptor coordinating methods for detection of CWAs. Lee *et al*. fabricated ultrafine carbon nanofibers coated with metal oxide by electrospinning a composite solution^3^. Lu *et al*. also reported DNA-decorated graphene chemiresistors in which DNA grafted on a clean and inert graphene sheet for the detection of organophosphonate (dimethyl methyl phosphonate, DMMP)^4^. So far, the mechanism of how sensing takes place in the presence of simulant have not been fully explained. Yasaei *et al*. studied the sensing properties of chemically untreated graphene at the grain boundaries^5^. They proved that the grain boundaries are highly sensitive sites for catching phosphonates by sequentially checking the numbers of graphene grain boundaries. They showed the sensitivity up to 300 times higher than that of a single graphene grain, but the fabrication of these devices is complicated, and a rapid response sensing system is still required. For these reasons, the development of novel sensing materials with facile processing, fast response and a dual checking system to reduce misdiagnoses is urgently required in sensor applications.

Many materials have been widely applied in sensing platforms such as metal oxides, organic semiconductors and carbon based substances. Graphene quantum dots (GQDs) have drawn considerable attention in this field because of their advantageous properties such as high surface to volume ratio, low toxicity, excellent electrical properties, easy solution processing, strong photo-luminescence and controllable band gap with functionalized molecules^6^,^7^,^8^,^9^. For example, GQD-based sensors have been designed for use in immuno-sensors, metal ion catchers, and humidity detectors^10^. However, to date, there are no reports on GQD based sensor related to the detection of nerve agents even though these chemicals are highly toxic due to their blocking properties, which occur in the body by phosphorylation of an active enzyme site^1^. As a receptor for DMMP, a hexafluorohydroxypropanyl benzene (HFHPB) functional group is well known as a strong acid and hydrogen bonding reagent. And there are many reports about hydrogen bonding interaction between hexafluorohydroxypropanyl group and basic compounds as quinone^11^. So it is strongly believed that the HFHPB moiety shows an advanced property on interaction with organophosphorus DMMP compound.

In this work, we report novel band gap-controlled HFHPB-GQD material and its chemical sensor. We carefully design new sensor system using GQD to provide the maximum change in the photoluminescence (PL) intensity. The main design concept for the sensor is to use the HFHPB functional molecule for quenching the fluorescence intensity of HFHPB-GQD by modulating the energy band gap and then recovering the fluorescence intensity of HFHPB-GQD by detecting DMMP to give an enhanced sensitivity and selectivity to DMMP in comparison with GQD without HFHPB. New HFHPB-GQD material is successfully prepared by covalent carbon-carbon bonding of HFHPB on the GQD *via* the diazonium coupling reaction. By breaking a conjugated π electron system through the covalent carbon-carbon bonding, its energy difference (δE) between σ and π orbitals is expected to be reduced and, as a result, the energy band gap of HFHPB-GQD is expected to be widened, compared with that of only GQD without HFHPB group. Specifically, the HFHPB functional group that has a strong electron withdrawing trifluoromethyl group is introduced into the GQD backbone to decrease the PL intensity and after addition of DMMP, the PL intensity is expected to be sharply recovered. In contrast, the PL intensity of the GQD without HFHPB does not change after exposure to the DMMP. We assume that the strong acidic proton produced by the electron withdrawing CF_3_ functional group of HFHPB-GQD can easily hydrogen-bond with phosphonate functional groups of DMMP. In addition, our designed HFHPB-GQD is expected to be selective to only the phosphonate functional group among many other analytes including organic and aqueous solvents. We also expect that our new HFHPB-GQDs would be very stable for real device applications since GQDs are very stable even under an oxygen atmosphere. Moreover, HFHPB-GQDs provide easy and simple solution processing for real device systems because of their excellent dispersion in most solvents. An illustration of our new HFHPB-GQD and its interaction with the target material (DMMP) was shown in Fig. 1.

## Results

### Characterization of HFHP-GQD

The HFHPB grafted GQD was characterized using transmission electron microscopy (TEM), and atomic force microscopy (AFM), Fourier transform infrared (FT-IR) spectroscopy, Raman spectroscopy, X-ray photoelectron spectroscopy (XPS), UV-vis spectroscopy (UV-vis), photoluminescence spectroscopy (PL) as shown Figs 2 and S1–S4. To protect from insertion of nitrogen and other additional oxygen functional groups before adding the grafted molecules, we synthesized bare GQDs using a solvothermal method with ethanol to obtain a clean and intrinsic GQD (see experimental section)^8^.

**Figure 1:**
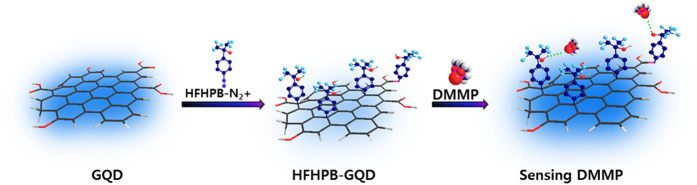
An illustration of our sensory material referred to as HFHPB grafted GQD (HFHPB-GQD) and its interactions with the target material (DMMP).

**Figure 2:**
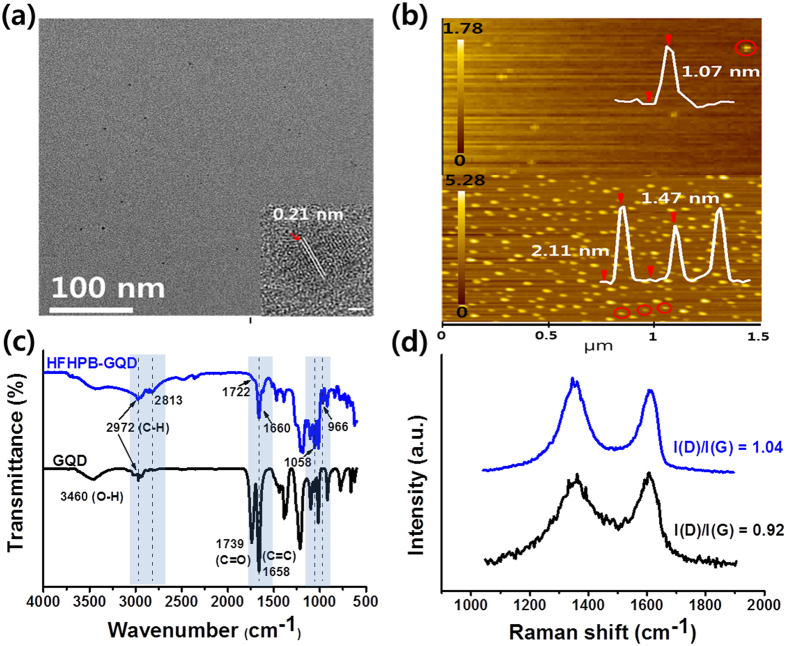
Characterization of HFHPB-GQDs. (**a**) TEM image, inset; HRTEM image of HFHPB-GQDs (scale bar 1 nm). (**b**) AFM image of GQD (top), HFHPB-GQD (bottom). (**c**) Fourier transform infrared (FTIR) spectra of GQDs (black) and HFHPB-GQDs (blue). (**d**) Raman spectra of GQDs (black) and HFHPB-GQDs (blue) on a SiO2/Si substrate with a 514 nm laser. The peak shift was calculated after Gaussian fitting.

Figure 2 shows transmission electron microscopy (TEM) images of HFHPB-GQD and as-prepared GQDs (Figure S1). As-prepared GQDs had an average diameter of 5 nm. The spacing of the lattice was measured to be on the order of 0.21 nanometers, which is related to the [110] planes of graphite (Figure S1 inset)^12^. The TEM images of modified GQDs are shown in Fig. 2a, and these images do not show a change in diameter. This result means that the functionalization did not affect the size of the GQDs. The high resolution TEM image shows highly crystalline with a lattice parameter of 0.21 nm even after grafting HFHPB (inset of Fig. 1a and Figure S2)^13^,^14^. Observation of the morphology and thickness of the HFHPB-GQDs and bare GQDs were conducted by AFM. As shown in Fig. 2b, individual HFHPB-GQDs were observed with an average thickness of 2 nm. This indicates that the HFHPB moiety was successfully grafted on the GQD platform.

HFHPB-GQDs were further characterized by FT-IR and Raman spectroscopy. The FT-IR spectra of GQDs showed a vibrational adsorption band of C=O stretching in COOR moieties at 1739 cm^−1^ and an O-H stretching vibration at 3460 cm^−1^. The typical peaks of C-H asymmetric and symmetric vibration (nearby 2972 cm^−1^) were observed in addition to a new peak (2813 cm^−1^), which is attributed to the stretching vibration of the C-H bond. In addition, vibrational C-H bending in-plane (1058 cm^−1^) and hydrogen wagging out of plane (966 cm^−1^) from the aromatic moieties in HFHPB were observed, which imply that HFHPB was attached to the GQDs^15^,^16^,^17^. Furthermore, the relative intensities of the peak at 1658 cm^−1^, which corresponds to the C=C stretching vibration, increased when the HFHPB moiety was integrated into GQD. This is because of an increased intensity in the aromatic region due to the introduction of an HFHPB-connected benzene moiety. And during the functionalization, as noted previously, the diazonium reaction involves decarboxylation of edge site of GQD through nucleophilic addition of the enolate anion to the diazonium salt produces the azo compound named by Japp–Klingemann reaction^18^.

Raman spectroscopy measurements also imply the formation of HFHPB-GQDs as shown in Fig. 1d. The typical GQD peaks of D and G were displayed at about 1365 cm^−1^ and 1601 cm^−1^, respectively^8^. In contrast to GQD, the D band of HFHPB-GQDs was shifted to lower wavenumbers by 11 cm^−1^ (1354 cm^−1^), and the intensity ratio of the D to the G band (*I*_*D*_/*I*_*G*_) was estimated to be about 1.04, which increased relative to bare GQD by about 0.92. These results indicate that structural disorder occurred in the GQD platform as previously observed by Jeon *et al*.^13^,^19^,^20^.

**Figure 3:**
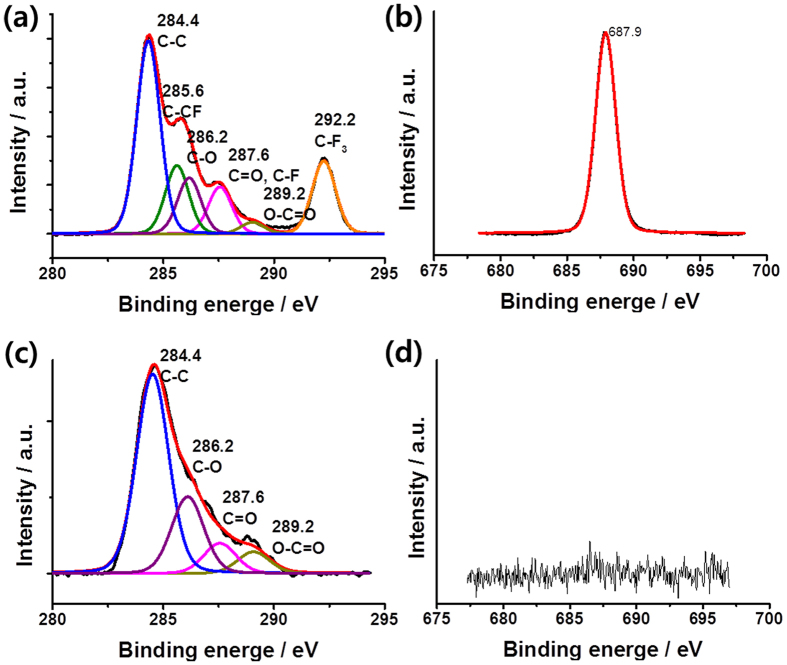
(**a,b**) X-ray photoelectron spectra of C 1 s and F 1 s for HFHPB-GQDs. (**c,d**) C1s and F1s for GQDs.

The HFHPB grafted GQDs were analyzed by XPS. The total amount of carbon, fluorine, oxygen and nitrogen were 45, 19, 30, and 6%, respectively, for HFHPB-GQD (Figs 3 and S2). The deconvoluted C1s spectra of HFHPB-GQD showed several components at 284.4, 285.6, 286.2, 287.6, and 289.2 eV, which were related to C-C, C-CF, C-O, C=O or C-F, and O-C=O, respectively^21^,^22^,^23^. A strong chemically shifted peak was observed at 292.2 eV, and this is related to the binding energy of C-F because the high electronegativity of fluorine resulted in the functionalization of GQDs with a HFHPB moiety^22^,^24^. Furthermore, the HFHPB-GQD showed a pronounced F 1 s peak with a F/C atomic ratio of 42.22%, while no F signal was observed for the bare GQD. These results indicate that the HFHPB moiety was stably coordinated on the GQD. As noted previously, the diazonium reaction involves an azo linkage in addition to the covalent carbon-carbon bond when phenoxy functional groups are located on the carbon surface^25^,^26^. Similarly, our HFHPB-GQD also had an azo-linked HFHPB moiety with no contribution from a high binding energy peak at 405 eV, which would be related to nitrogen in a diazonium group^26^.

**Figure 4:**
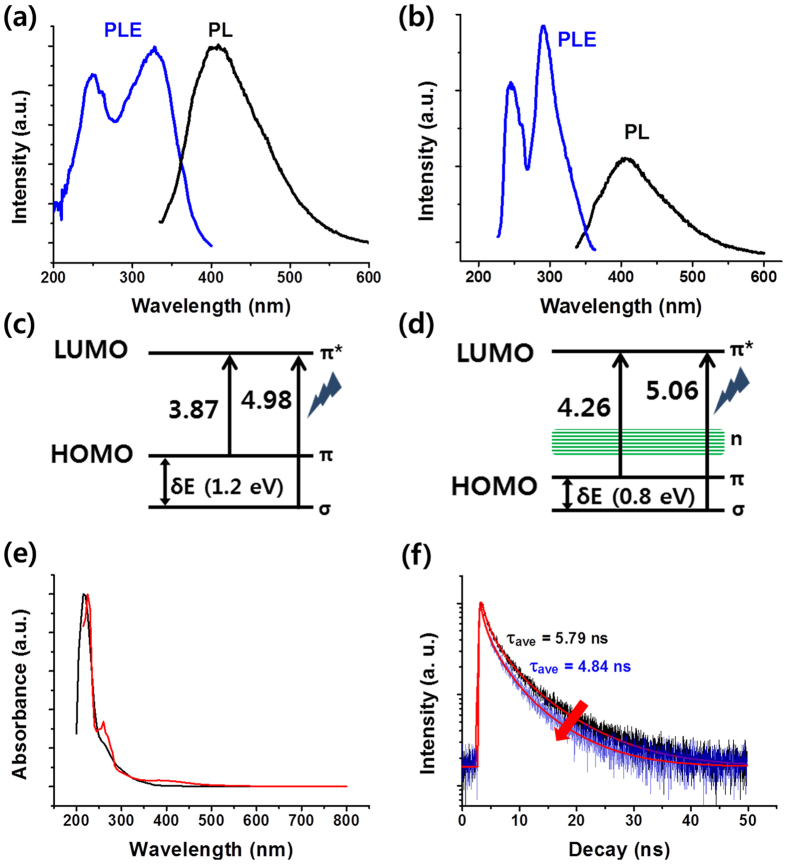
Optical properties of GQDs and HFHPB-GQDs. Photoluminescence excitation (PLE) (blue) and PL (black) spectra of (**a**) GQD, (**b**) HFHPB-GQD. Energy band diagram of (**c**) GQD, (**d**) HFHPB-GQD. (**e**) UV-vis spectra of GQD (black) and HFHPB-GQD (red). (**f**) Time resolved fluorescence decay curves of GQD (black) and HFHPB-GQD (blue) (λ_ex_ = 340 nm, λ_em_ = 405 nm).

The optical properties of GQD and HFHPB-GQD were investigated by UV-vis absorption spectroscopy and PL spectroscopy. The PL excitation (PLE) spectra shown in Fig. 4a and b were obtained to further examine the recombination phenomena answerable for the emission. Two electronic transitions of bare GQDs were detected at 249 nm (4.98 eV) and 328 nm (3.78 eV) at λ_em_ 404. Theses peaks are regarded as the two sorts of the electronic transition from σ and π molecular orbitals to the lowest unoccupied molecular orbital (LUMO) as shown in Fig. 4c and d. In the case of HFHPB-GQD, 245 nm (5.06 eV) and 291 nm (4.26 eV) were observed for the transition of σ and π orbitals to LUMO^27^,^28^. According to the previous reports^29^, the energy difference (δE) between σ and π orbitals should be below 1.5 eV for a triple ground state. The energy difference of GQD and HFHPB-GQD have been calculated to be 1.2 eV and 0.8 eV, respectively, which are probable value for triplet ground state of carbene (<1.5 eV). Thus, the HFHPB functional group can modulate the δE between σ and π orbitals.

As seen in Fig. 4e, both GQDs and HFHPB-GQDs show strong absorption peaks in the deep UV (<250 nm), which are assigned to π-π* transitions of aromatic C=C bonds according to a previous report^30^,^31^. An additional shoulder peak at 265 nm was assigned to the n-π *electron transition of heteroatoms (C=O) existed in GQDs. The absorption peak for the n-π * transition was changed from 265 nm to 260 nm with an absorption at 395 nm, which is related to new transition site (O or N related interstate band) of HFHPB-GQD^32^. Due to the oxygen containing group in HFHPB, the oxygen or HFHPB related surface state may be induced between π band and π* band in HFHPB-GQD. The fluorescent properties are originated from the π conjugated system of the carbon domain on GQD due to π-π electron transitions. The PL intensity of the GQD sharply decreased after introducing the HFHPB moiety onto the GOD in comparison with the same concentration of unmodified GQDs as shown in Fig. 3a and b. This result means that sp^2^ carbon atoms of the aromatic rings were involved in carbon addition to form sp^3^ hybridization through embedding HFHPB moieties on the GQD; this result reflects the introduction of electron withdrawing trifluoromethyl groups as reported previously^13^,^22^. In addition, new appearance of interstate band through HFHPB group in band gap may also affect in decreasing PL intensity. Moreover, according to a previous report, graphene and other carbon materials functionalized with fluorine showed a large blue shift in UV-vis and PL spectra as a result of the band gap widening caused by the polarization-induced charge effect^24^. Consequently, the emission peak was shifted from 412 nm to 407 nm, which is consistent with the behavior of our UV-vis spectra^31^. Our UV-vis and PL spectra showed that the blue shift phenomena increased and the PL intensity decreased as the amount of HFHPB diazonium salt on a fixed amount of GQD increased (Figure S4). The PL decay of GQD and HFHPB-GQD are measured by a time correlated single photon counting technique with fitting by triple exponential function at 375 nm excitation as shown in Fig. 4f. The observed life time of GQD is τ_ave_ = 5.79 ns, whereas that of HFHPB-GQD is τ_1_ = 4.84 ns, showing that HFHPB-GQD is shorter decay time than GQD due to the electron withdrawing HFHPB group. For the measurement, the ratio (0.5 vs 1 with weight ratio) of HFHPB-N2^+^ (receptor) *versus* GQD was fixed and the same excitation wavelength to investigate the sensing properties of HFHPB-GQD with PL spectra was observed.

### Sensing property of HFHPB-GQD to DMMP

**Figure 5:**
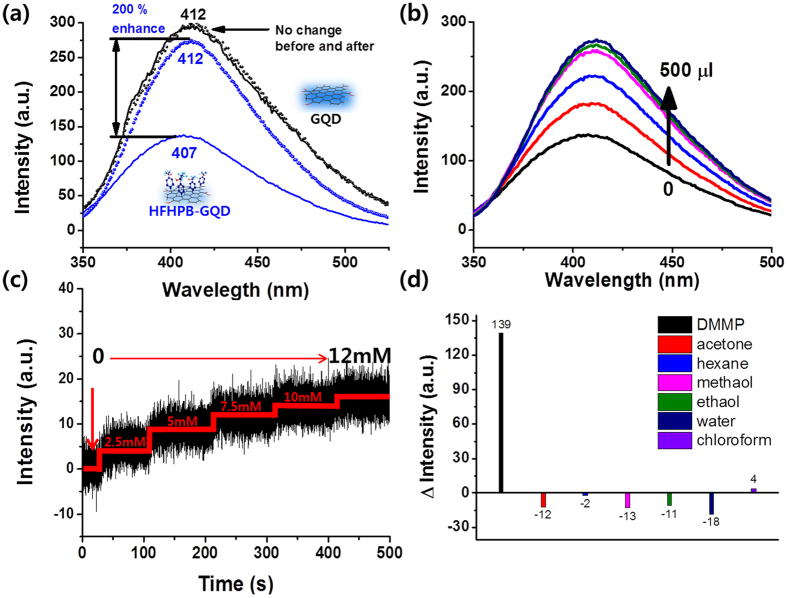
(**a**) Photoluminescence spectra. Solid line - 2 mg/ml of GQDs (black) and HFHPB-GQDs (blue) and circle line – after adding of 500 μl of DMMP. (**b**) Sensing properties of HFHPB-GQDs for 100 μl increases in the amount of 0.5 M DMMP. (**c**) Time dependent photoluminescence spectra for HFHPB-GQDs by introducing various concentrations of DMMP. (**d**) Selectivity test: photoluminescence change of HFHPB-GQDs on injection of 500 μl of organic volatile solvents and DMMP. Excitation wavelength was at 330 nm.

The sensing response of HFHPB-GQD was monitored by PL spectroscopy and a quartz crystal resonator to conduct a quantitative analysis. Figure 5 represents the fluorescent sensing behavior of GQDs and HFHPB-GQDs in response to DMMP. To precisely determine the sensing properties, both GQD and HFHPB-GQD were dissolved in dichloromethane to reduce the interaction between solvent and substances before exposure to DMMP. Generally, the excited electrons can emit PL light through radiative recombination. However, the HFHPB may contribute as non-emissive trap state between π band and π* band. Therefore, the PL intensity of HFHPB-GQDs dramatically decreased and a blue-shift phenomenon was observed from 412 to 407 nm as shown in Fig. 5a. Interestingly, by adding 500 μl of 0.5 M of DMMP, the PL intensity of the HFHPB-GQDs (blue line) sharply increased (by about 200%) as compared with bare GQD (black line) and exhibited a red shift in wavelength, recovering back to the original wavelength, 412 nm (open circle line). We assume that the decreased radiative recombination through the non-emissive trap state from HFHPB functionality could be increaseed *via* hydrogen bonding with analyte (DMMP). Thus, the PL intensity is returned back to the original value. Figure 5b showed a gradual increase in PL intensity and the maximum wavelength peaks by adding 100 μl of the same concentrated DMMP solution. The changes in PL intensity and maximum emission wavelength of HFHPB-GQD with DMMP are considered to be the result of proton induced charge transfer *via* hydrogen bonding and the introduction of scattering sites as noted in a previous report^33^. The total enhanced fluorescence was 200% compared with initial response of HFHPB-GQD. The PL lifetime of GQD, HFHPB-GQD and HFHPB-GQD interacted with DMMP were also examined as shown in Table S1 and Figure S5. As HFHPB functional group provides the non-emissive trap site to GQD after grafting HFHPB functionality, we explained earlier, the decay life time of PL was diminished from τ_ave_ = 5.79 ns to τ_ave_ = 4.84 ns. Interestingly, the decay lifetime of HFHPB-GQD was enhanced after adding DMMP (τ_ave_ = 5.05 ns). The results of average PL decay life time could be matched with the sensing behavior in PL (Fig. 5a). On the other hand, GQD without the HFHPB moiety showed no significant changes in fluorescence even with equal concentrations of DMMP. In partial conclusion, this result implies that our newly designed sensor platform using HFHPB-GQD is extremely effective for detecting a specific nerve agent and will guide the development of other specific sensing materials and devices in the future.

To clearly understand a hydrogen bonding interaction between HFHPB moiety and DMMP, we carefully performed ^1^H and ^31^P NMR experiments as shown in Fig. S6. For a control experiment, ^1^H NMR spectra of DMMP and HFHPB-NH_2_ were taken as shown in Fig. S6a and b, respectively. Figure S6C showed ^1^H NMR spectrum for a mixture of DMMP and HFHPB-NH_2_. According to literature, the acidic hydroxylic proton caused by electron withdrawing CF_3_ groups of HFHPB-NH_2_ makes easily a strong hydrogen bonding with basic compounds^34^. As a result of a strong hydrogen bonding interaction of HFHPB-NH_2_ with phosphonate group of DMMP, a large diamagnetic shift of the hydroxyl proton was occurred, showing that the hydroxyl proton (OH) of HFHPB-NH_2_ was shifted from 3.36 ppm to 4.51 ppm. In addition, an experiment of hydrogen deuterium exchange was investigated as seen in Fig. S6d. After adding 1 drop of D_2_O in the tube of mixed DMMP and HFHPB-NH_2_, the hydroxyl proton was disappeared, which means that the hydroxyl proton was exchanged with deuterium of D_2_O. All these results strongly supported our photoluminescence data, indicating that the HFHPB receptor functionalized on GQDs can affect largely for detecting organophosphonate of DMMP through the noticeable change on photoluminescence^35^,^36^. On the other hand, ^31^P NMR of DMMP^37^ and a mixture of DMMP with HFHPB-NH_2_ showed no big change in the chemical shift as exhibited in Fig. S6e and f.

For more precise determination of a sensing limit in the liquid state, time resolved PL was conducted. Figure 5c demonstrates the time dependent integrated PL intensity of HFHPB plugged GQDs upon exposure to different concentrations of DMMP solutions at a fixed emission of 410 nm. The gradual increase in PL intensity of HFHPB-GQD was observed after adding various concentrations of DMMP. The detection limit of the HFHPB-GQD was about 2.5 mM of DMMP, which is identified from the time resolved photoluminescence spectroscopy in the liquid state. In addition, an investigation of selectivity was done by PL spectroscopy in the presence of assorted analytes at identical ranges of fixed wavelength on emission as shown in Fig. 5d. HFHPB-GQD showed outstanding selectivity for recognizing DMMPs among other organic solvents, which means that the HFHPB moiety allows the GQDs to be a perfect indicator of DMMP.

**Figure 6:**
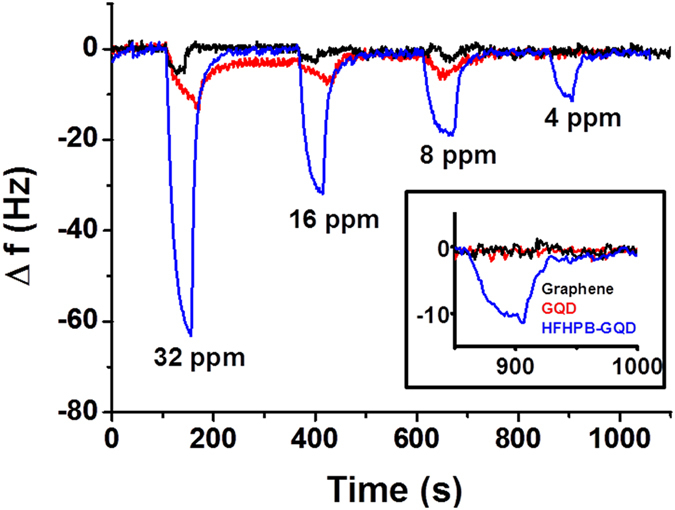
Nanogravimetry response of HFHPB-GQDs coated on QCM while releasing DMMP. Inset: Magnified frequency change of HFHPB-GQDs at 4 ppm.

We further investigated the quantitative adsorption of gaseous DMMP on the surface of HFHPB-GQD using a quartz crystal microbalance (QCM) as shown in Fig. 6. The solution of HFHPB-GQD was drop-casted onto gold electrodes of QCM. We used a mass flow controller (MFC) to control the flow of gaseous DMMP as explained in the Supporting Information. The whole MFC system was connected to the QCM in order to check the sensing response. Figure 6 showed a sensitivity of HFHPB-GQD to DMMP *via* the hydrogen bonding interactions on QCM. As shown, the frequency of the quartz crystal resonator changed based on the concentration of gaseous DMMP. An uncoated bare electrode of quartz crystal was tested as a control (Figure S7). The frequency of the bare quartz crystal had constant frequency as a function of time upon exposure to DMMP from 32 to 4 ppm, which means that the DMMP had no effect on the bare gold coated quartz crystal. In order to examine the adsorption and desorption time for DMMP, three different samples including cleaned graphene, non-treated GQDs, and HFHPB-GQDs were used as shown in Fig. 6. In case of GQD, it may have interaction between hydroxyl group and DMMP due to its oxygen containing functional groups. Thus GQD shows small portion of adsorption and desorption. Only the HFHPB-GQD coated quartz crystal showed a significant sharp change in frequency with regard to the adsorption and desorption as a function of time. After exposure, the active layers were purged with N_2_ to return them to their initial conditions. Finally, we found that DMMP adsorbed at masses of 39.78 × 10^−9^ g at 32 ppm, 20.08 × 10^−9^ g at 16 ppm, 11.97 × 10^−9^ g at 8 ppm, and 6.84 × 10^−9^ g at 4 ppm on HFHPB-GQDs coated quartz crystals. These results were obtained by calculation using the frequency change (Δƒ) in the Sauerbrey equation^38^. The change in the frequency was fully reversible, and the initial frequency value was recovered by purging with N_2_ for 34 s after shutting off the DMMP gas supply. The change in frequency increases as the concentration of DMMP increases, which means that the change in frequency is proportional to the concentration of gaseous DMMP. Surprisingly, our results are comparable to the limit of detection in chemiresistor sensors with modified graphene, functionalized carbon nanotubes, and polymer integrated reduced graphene oxide^4^,^39^,^40^. In order to analyze the times required for response and recovery, changes in the magnified frequencies versus time at 4 ppm were plotted for all samples in Fig. 6 inset. The average time to approach 90% adsorption of DMMP is 31 s for HFHPB-GQD, while those of graphene and non-treated GQD showed no response to exposure of gaseous DMMP at 4 ppm, which means that the HFHPB-GQD was successfully fabricated and sensitivity was improved by addition of the HFHPB moiety. Furthermore, the response time and the recovery time are 31 and 34 seconds, respectively, which is much shorter than those of graphene and bare GQDs (Figure S8, Table S2). The fast response and fast recovery in comparison with the bare graphene and GQDs indicate that the receptor (terminal HFHPB group) was well organized on the GQD platform and it effectively captured the gaseous DMMP.

It is obvious that the HFHPB-GQD was strongly interacted with DMMP by hydrogen boding which is the interaction between acidic proton and basic compound as we designed. The result indicates that the GQD modified with HFHPB moiety has the high sensitivity and selectivity than neat graphene and GQD against DMMP vapor and liquid. Our novel functionalized GQD will provide a promising direction in the development of carbon based sensor platforms.

## Discussion

In summary, for tuning a band gap of the photoluminescent GQD, an electron withdrawing HFHPB group was chemically attached on the GQD through covalent carbon-carbon bonding of HFHPB on the GQD by the diazonium coupling reaction of HFHPB diazonium salt, providing a novel HFHPB-GQD material that can be eligible for detecting a chemical warfare agent, DMMP. With a help of the electron withdrawing HFHPB group, the energy band gap of new HFHPB-GQD was widened and its PL decay life time decreased. As a result, the PL intensity of HFHPB-GQD dramatically decreased and the blue-shift phenomenon was observed from 412 to 407 nm wavelength. After addition of DMMP, however, the PL intensity of HFHPB-GQD sharply increased (by about 200%) through a hydrogen bond with DMMP that was confirmed with nanogravimetry measurement and H NMR study, and its maximum PL peak was moved back to the original wavelength, 412 nm, which showed a fast response and short recovery time. Thus, our HFHPB-GQD sensor shows high sensitive to DMMP in comparison with sensors with GQD without HFHPB and graphene. In addition, the HFHPB-GQD sensor exhibited high selectivity only to the phosphonate functional group among many other analytes including organic and aqueous solvents and also stable enough for real device applications. This break-through band gap tuning of GQD using electron withdrawing functional molecules may open up a promising new direction in the engineering of molecules on target substrates for the enhancement of their electrical and optical properties.

## Methods

### Materials and characterization

As synthesized GQDs and HFHPB-GQDs were characterized by atomic force microscopy (AFM, Agilent 5,100 AFM/SPM system), transmission electron microscopy (TEM, JEOL JEM-2100 Field Emission Gun HR-TEM), Fourier transform infrared spectroscopy (FT-IR, Bruker Vertex 70), and Raman spectroscopy (WiTec, Alpha 300 R with a 532-nm laser). X-ray photoelectron spectroscopy (XPS) measurements were taken in a SIGMA PROBE (ThermoVG) using a monochromatic Al-Kα X-ray source at 100 W. Photoluminescence (PL) spectra were obtained on a Cary eclipse fluorescence spectrophotometer (Agilent, Technologies, America). The sensing properties of GQDs and HFHPB-GQDs were monitored by a nanogravimetry technique (EQCN, Shin) using a quartz crystal resonator (formed on a gold-coated quartz crystal from International Crystal Manufacturing Co, Inc.). All measurements were carried out at room temperature (25 °C). Fluorescence lifetime decay was measured using a confocal microscope (MicroTime-200, Picoquant, Germany) with a 20x objective. A pulsed diode laser (375 nm with a pulse width of ~240 ps and an average power of ~1 μW) was used as an excitation source. An avalanche photodiode detector (PDM series, MPD) was used to collect whole emissions from the samples. Time-correlated single-photon counting (TCSPC) technique was used to count fluorescence photons^41^.

### Synthesis of GQDs

Graphene oxide was synthesized by a modified hummers method. Graphene quantum dots were obtained by a solvent assisted thermal reduction of graphene oxide. Specifically, 2 mg∙ml^−1^ of graphene oxide was placed in ethanol and then the solution was sonicated for 1 day. After sonication, the solution was poured into an autoclave to provide heat treatment in a muffle furnace at 200 °C for 20 h. The autoclave was cooled down to room temperature after the thermal treatment. Then, the yellowish solution was filtered. The solvent of the yellowish solution was evaporated for further chemical treatment.

### Synthesis of 4-(1,1,1,3,3,3-hexafluoro-2-hydroxypropan-2-yl)benzene diazonium tetrafluoroborate salt (HFHPB-N2^+^)

The diazonium salt of HFHPB-N2^+^ was synthesized according to a previous report^42^. 2-(4-Aminophenyl)-1,1,1,3,3,3-hexafluoro-2-propanol (0.200 g, Alfa Aesar) was dissolved in a 50 ml round bottom flask with dry THF (5 ml) under a N_2_ atmosphere. This solution was added dropwise to boron trifluoride etherate (2 equiv.) in a round bottom flask cooled to −5 °C under N_2_. Isoamyl nitrite (1.6 equiv.) was then added dropwise to the mixture with a syringe, and a solid subsequently formed. The mixture was allowed to stir for 30 min at −5 °C before it was allowed to return to room temperature. The solid was then filtered, and the filtered white power was washed with small amount of cold Et_2_O to yield a white solid after drying: characterization data for hexafluoroisopropylbenzenediazonium tetrafluoroborate; ^1^H NMR (500 MHz, CO(CD_3_)_2_, δ), ppm δ = 8.53 (d, Ar-H, 2H), 9.09 (d, Ar-H, 2H).

### Synthesis of HFHPB-GQD

GQDs were dissolved completely into ethanol to make a 10 mg∙ml^−1^ solution, which was stirred for a few minutes. The synthesized HFHPB-N2^+^ was dissolved into the solution of GQDs. The solution was stirred in a sealed container in the dark overnight. To remove the impurities and unexpected starting materials, the solution was dialyzed using a dialysis bag (retained molecular weight: 1000 Da) for 1 day.

### Mass flow controller (MFC) settings

A mass flow controller (MFC) system equipped with a N_2_ gas and bubbler system for the liquid was utilized for sensing the DMMP vapor. Dry N_2_ was used as the carrier gas. A certain amount of liquid DMMP was placed into the bubbler chamber, and the carrier gas was continuously passed through the headspace of the bubbler system. The carrier gas was controlled using an individual flow controller. The concentration was controlled using a regulating valve connected to the bubbler to obtain a fixed carrier gas flow rate.

## Additional Information

**How to cite this article**: Hwang, E. *et al*. Chemically modulated graphene quantum dot for tuning the photoluminescence as novel sensory probe. *Sci. Rep.* **6**, 39448; doi: 10.1038/srep39448 (2016).

**Publisher's note:** Springer Nature remains neutral with regard to jurisdictional claims in published maps and institutional affiliations.

## Supplementary Material

Supplementary Information

## References

[b1] Kim K., Tsay O. G., Atwood D. A. & Churchill D. G. Destruction and Detection of Chemical Warfare Agents. Chemical Reviews 111, 5345–5403 (2011). 21667946 10.1021/cr100193y

[b2] Darvish Ganji M., Dalirandeh Z., Khosravi A. & Fereidoon A. Aluminum nitride graphene for DMMP nerve agent adsorption and detection. Materials Chemistry and Physics 145, 260–267 (2014).

[b3] Coleman J. N. . Two-Dimensional Nanosheets Produced by Liquid Exfoliation of Layered Materials. Science 331, 568–571 (2011). 21292974 10.1126/science.1194975

[b4] Lu Y., Goldsmith B. R., Kybert N. J. & Johnson A. T. C. DNA-decorated graphene chemical sensors. Applied Physics Letters 97, 083107 (2010).

[b5] Yasaei P. . Chemical sensing with switchable transport channels in graphene grain boundaries. Nat Commun 5 (2014). 10.1038/ncomms5911 25241799

[b6] Shen J., Zhu Y., Yang X. & Li C. Graphene quantum dots: emergent nanolights for bioimaging, sensors, catalysis and photovoltaic devices. Chemical Communications 48, 3686–3699 (2012). 22410424 10.1039/c2cc00110a

[b7] Bacon M., Bradley S. J. & Nann T. Graphene Quantum Dots. Particle & Particle Systems Characterization 31, 415–428 (2014).

[b8] Li L. . Focusing on luminescent graphene quantum dots: current status and future perspectives. Nanoscale 5, 4015–4039 (2013). 23579482 10.1039/c3nr33849e

[b9] Liu Y., Dong X. & Chen P. Biological and chemical sensors based on graphene materials. Chemical Society Reviews 41, 2283–2307 (2012). 22143223 10.1039/c1cs15270j

[b10] Sun H., Wu L., Wei W. & Qu X. Recent advances in graphene quantum dots for sensing. Materials Today 16, 433–442 (2013).

[b11] Gupta N. & Linschitz H. Hydrogen-Bonding and Protonation Effects in Electrochemistry of Quinones in Aprotic Solvents. Journal of the American Chemical Society 119, 6384–6391 (1997).

[b12] Tang L. . Deep Ultraviolet Photoluminescence of Water-Soluble Self-Passivated Graphene Quantum Dots. ACS Nano 6, 5102–5110 (2012). 22559247 10.1021/nn300760g

[b13] Jeon K.-J. . Fluorographene: A Wide Bandgap Semiconductor with Ultraviolet Luminescence. ACS Nano 5, 1042–1046 (2011). 21204572 10.1021/nn1025274

[b14] Johns J. E. & Hersam M. C. Atomic Covalent Functionalization of Graphene. Accounts of Chemical Research 46, 77–86 (2013). 23030800 10.1021/ar300143e PMC3546170

[b15] Luo P., Ji Z., Li C. & Shi G. Aryl-modified graphene quantum dots with enhanced photoluminescence and improved pH tolerance. Nanoscale 5, 7361–7367 (2013). 23824213 10.1039/c3nr02156d

[b16] Lu Q., Zhang Y. & Liu S. Graphene quantum dots enhanced photocatalytic activity of zinc porphyrin toward the degradation of methylene blue under visible-light irradiation. Journal of Materials Chemistry A 3, 8552–8558 (2015).

[b17] Lu Y. . Novel blue light emitting graphene oxide nanosheets fabricated by surface functionalization. Journal of Materials Chemistry 22, 2929–2934 (2012).

[b18] Phillips R. R. In Organic Reactions (John Wiley & Sons, Inc., 2004).

[b19] Tetsuka H. . Optically Tunable Amino-Functionalized Graphene Quantum Dots. Advanced Materials 24, 5333–5338 (2012). 22833282 10.1002/adma.201201930

[b20] Jin S. H., Kim D. H., Jun G. H., Hong S. H. & Jeon S. Tuning the Photoluminescence of Graphene Quantum Dots through the Charge Transfer Effect of Functional Groups. ACS Nano 7, 1239–1245 (2013). 23272894 10.1021/nn304675g

[b21] Zhu S. . Surface Chemistry Routes to Modulate the Photoluminescence of Graphene Quantum Dots: From Fluorescence Mechanism to Up-Conversion Bioimaging Applications. Advanced Functional Materials 22, 4732–4740 (2012).

[b22] Feng Q. . Synthesis and photoluminescence of fluorinated graphene quantum dots. Applied Physics Letters 102, 013111 (2013).

[b23] Mattiuzzi A. . Electrografting of calix[4]arenediazonium salts to form versatile robust platforms for spatially controlled surface functionalization. Nat Commun 3, 1130 (2012). 23072800 10.1038/ncomms2121

[b24] Robinson J. T. . Properties of Fluorinated Graphene Films. Nano Letters 10, 3001–3005 (2010). 20698613 10.1021/nl101437p

[b25] Mahouche-Chergui S., Gam-Derouich S., Mangeney C. & Chehimi M. M. Aryl diazonium salts: a new class of coupling agents for bonding polymers, biomacromolecules and nanoparticles to surfaces. Chemical Society Reviews 40, 4143–4166 (2011). 21479328 10.1039/c0cs00179a

[b26] Doppelt P., Hallais G., Pinson J., Podvorica F. & Verneyre S. Surface Modification of Conducting Substrates. Existence of Azo Bonds in the Structure of Organic Layers Obtained from Diazonium Salts. Chemistry of Materials 19, 4570–4575 (2007).

[b27] Vempati S. & Uyar T. Fluorescence from graphene oxide and the influence of ionic, [small pi]-[small pi] interactions and heterointerfaces: electron or energy transfer dynamics. Physical Chemistry Chemical Physics 16, 21183–21203 (2014). 25197977 10.1039/c4cp03317e

[b28] Deng X. . The emission wavelength dependent photoluminescence lifetime of the N-doped graphene quantum dots. Applied Physics Letters 107, 241905 (2015).

[b29] Moon B. J. . Facile and Purification-Free Synthesis of Nitrogenated Amphiphilic Graphitic Carbon Dots. Chemistry of Materials (2016).

[b30] Fan Z. . Surrounding media sensitive photoluminescence of boron-doped graphene quantum dots for highly fluorescent dyed crystals, chemical sensing and bioimaging. Carbon 70, 149–156 (2014).

[b31] Ju J. & Chen W. Synthesis of highly fluorescent nitrogen-doped graphene quantum dots for sensitive, label-free detection of Fe (III) in aqueous media. Biosensors and Bioelectronics 58, 219–225 (2014). 24650437 10.1016/j.bios.2014.02.061

[b32] Gupta A. & Saha S. K. Emerging photoluminescence in azo-pyridine intercalated graphene oxide layers. Nanoscale 4, 6562–6567 (2012). 22968198 10.1039/c2nr31891a

[b33] Wang F., Gu H. & Swager T. M. Carbon Nanotube/Polythiophene Chemiresistive Sensors for Chemical Warfare Agents. Journal of the American Chemical Society 130, 5392–5393 (2008). 18373343 10.1021/ja710795k

[b34] Middleton W. J. & Lindsey R. V. Hydrogen Bonding in Fluoro Alcohols. Journal of the American Chemical Society 86, 4948–4952 (1964).

[b35] Kong L. . Novel pyrenehexafluoroisopropanol derivative-decorated single-walled carbon nanotubes for detection of nerve agents by strong hydrogen-bonding interaction. Analyst 135, 368–374 (2010). 20098772 10.1039/b920266h

[b36] Zheng Q., Fu Y.-c. & Xu J.-q. Advances in the chemical sensors for the detection of DMMP — A simulant for nerve agent sarin. Procedia Engineering 7, 179–184 (2010).

[b37] Gordon M. D. & Quin L. D. Temperature dependence of 31P NMR chemical shifts of some trivalent phosphorus compounds. Journal of Magnetic Resonance (1969) 22, 149–153 (1976).

[b38] Seo S., Min M., Lee S. M. & Lee H. Photo-switchable molecular monolayer anchored between highly transparent and flexible graphene electrodes. Nat Commun 4, 1920 (2013). 23715279 10.1038/ncomms2937

[b39] Hu N. . Gas sensor based on p-phenylenediamine reduced graphene oxide. Sensors and Actuators B: Chemical 163, 107–114 (2012).

[b40] Saetia K. . Spray-Layer-by-Layer Carbon Nanotube/Electrospun Fiber Electrodes for Flexible Chemiresistive Sensor Applications. Advanced Functional Materials 24, 492–502 (2014).

[b41] Shin Y., Park J., Hyun D., Yang J. & Lee H. Generation of graphene quantum dots by the oxidative cleavage of graphene oxide using the oxone oxidant. New Journal of Chemistry 39, 2425–2428 (2015).

[b42] Fahrenholtz K. E. . 3-Phenyl-5-[2,2,2-trifluoro-1-hydroxy-1-(trifluoromethyl)ethyl]indole-2-carbonitrile, a potent inhibitor of prostaglandin synthetase and of platelet aggregation. Journal of Medicinal Chemistry 22, 948–953 (1979). 114655 10.1021/jm00194a012

