# Supplementary material for: Chemically modulated graphene quantum dot for tuning the photoluminescence as novel sensory probe
Source: Sci Rep. 2016 Dec 19;6:39448. doi: 10.1038/srep39448 (PMC5171703; doi:10.1038/srep39448)
Supplement: Supplementary Information [file srep39448-s1.doc]

Supporting Information

**Chemically modulated graphene quantum dot for tuning the photoluminescence as novel sensory probe**

*Eunhee Hwang^1,2^, Hee Min Hwang^1,3^, Yonghun Shin^1,3^, Yeoheung Yoon^1,3^, Hanleem Lee^1,3^, Junghee Yang^2^, Sora Bak^1,2^ and Hyoyoung Lee^1,2,3*^*

^1^Centre for Integrated Nanostructure Physics (CINAP), Institute of Basic Science (IBS), 2066 Seoburo, Jangan-gu, Suwon 16419, Republic of Korea

^2^Department of Chemistry, Sungkyunkwan University, 2066 Seoburo, Jangan-gu, Suwon 16419, Republic of Korea

^3^Department of Energy Science, Sungkyunkwan University, 2066 Seoburo, Jangan-gu, Suwon 16419, Republic of Korea

*Corresponding author. E-mail: [hyoyoung@skku.edu](mailto:hyoyoung@skku.edu); Fax: +82-031-290-5934; Tel: +82-031-299-4566.


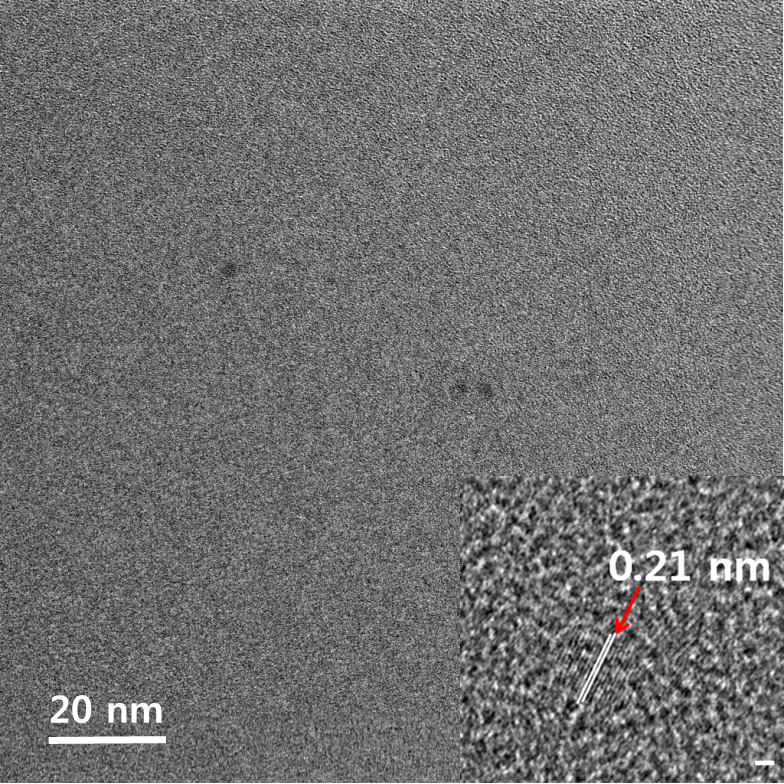


**Figure S1.** HRTEM image of GQDs by solvothermal treatment.

**
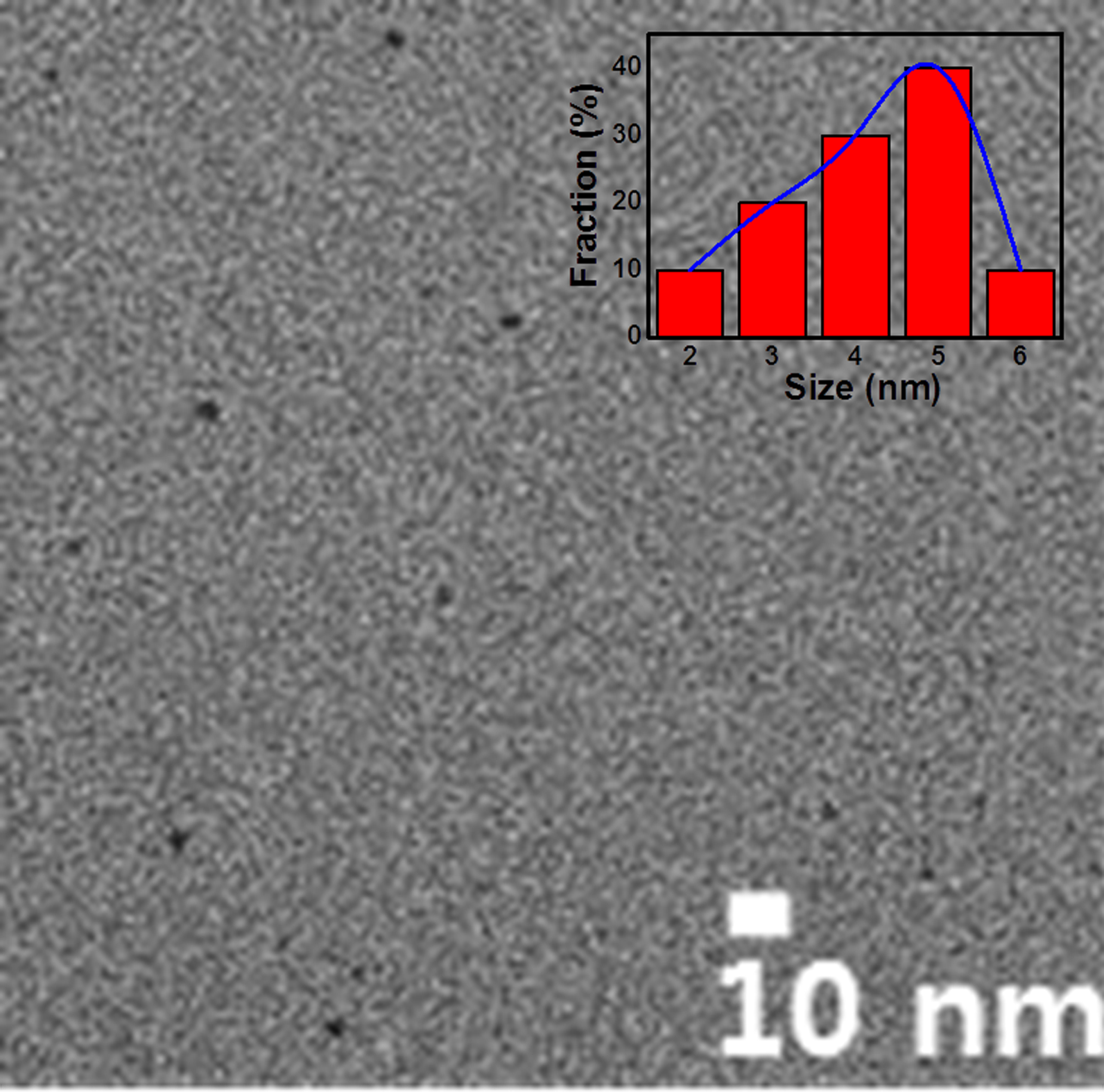
**

**Figure S2.** HRTEM image of HFHPB-GQDs with size distribution.


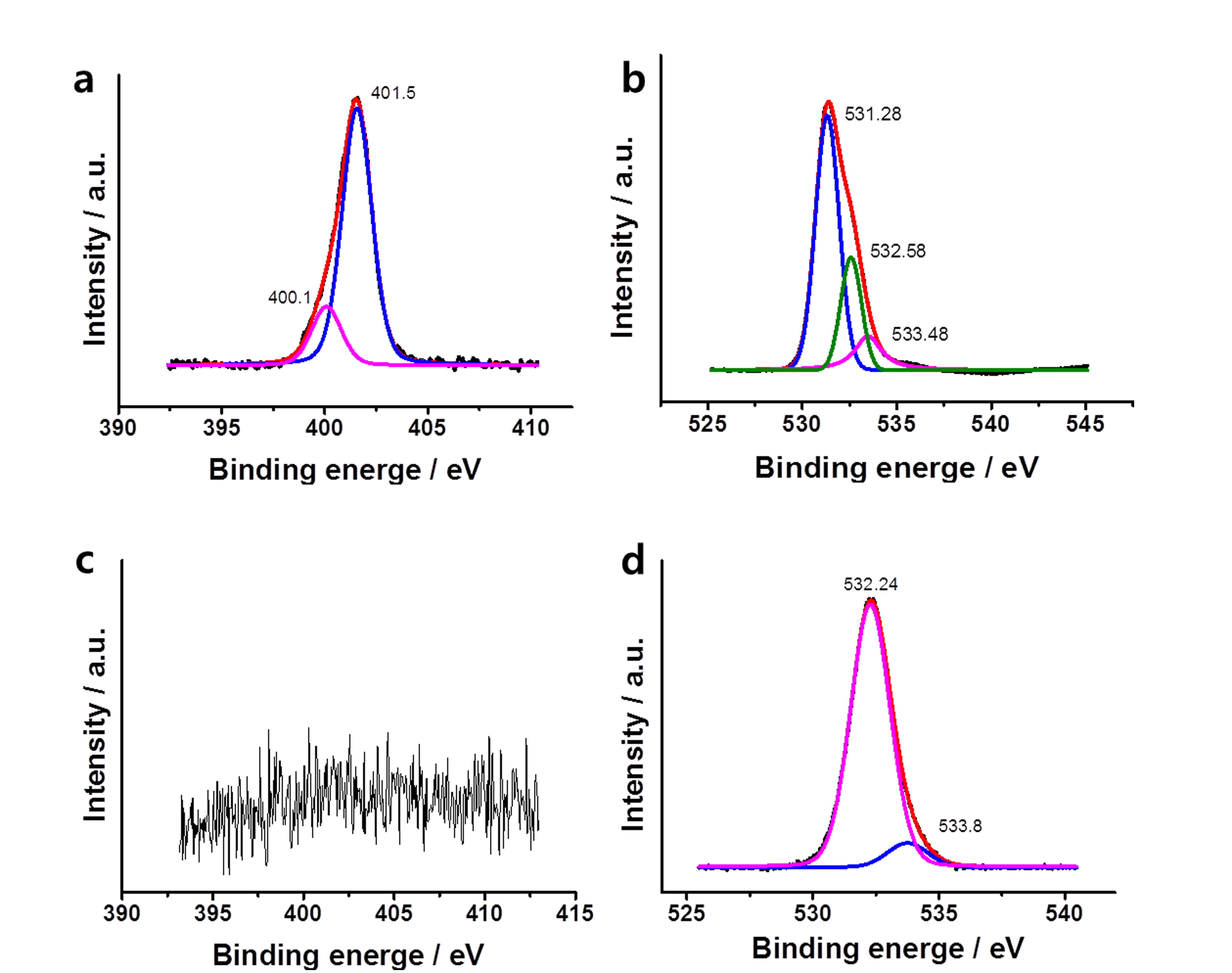


**Figure S3.** (a) (b) X-ray photoelectron spectra of N 1s and O 1s for HFHPB-GQDs. (c) (d) N 1s and O 1s spectra for GQDs**.**


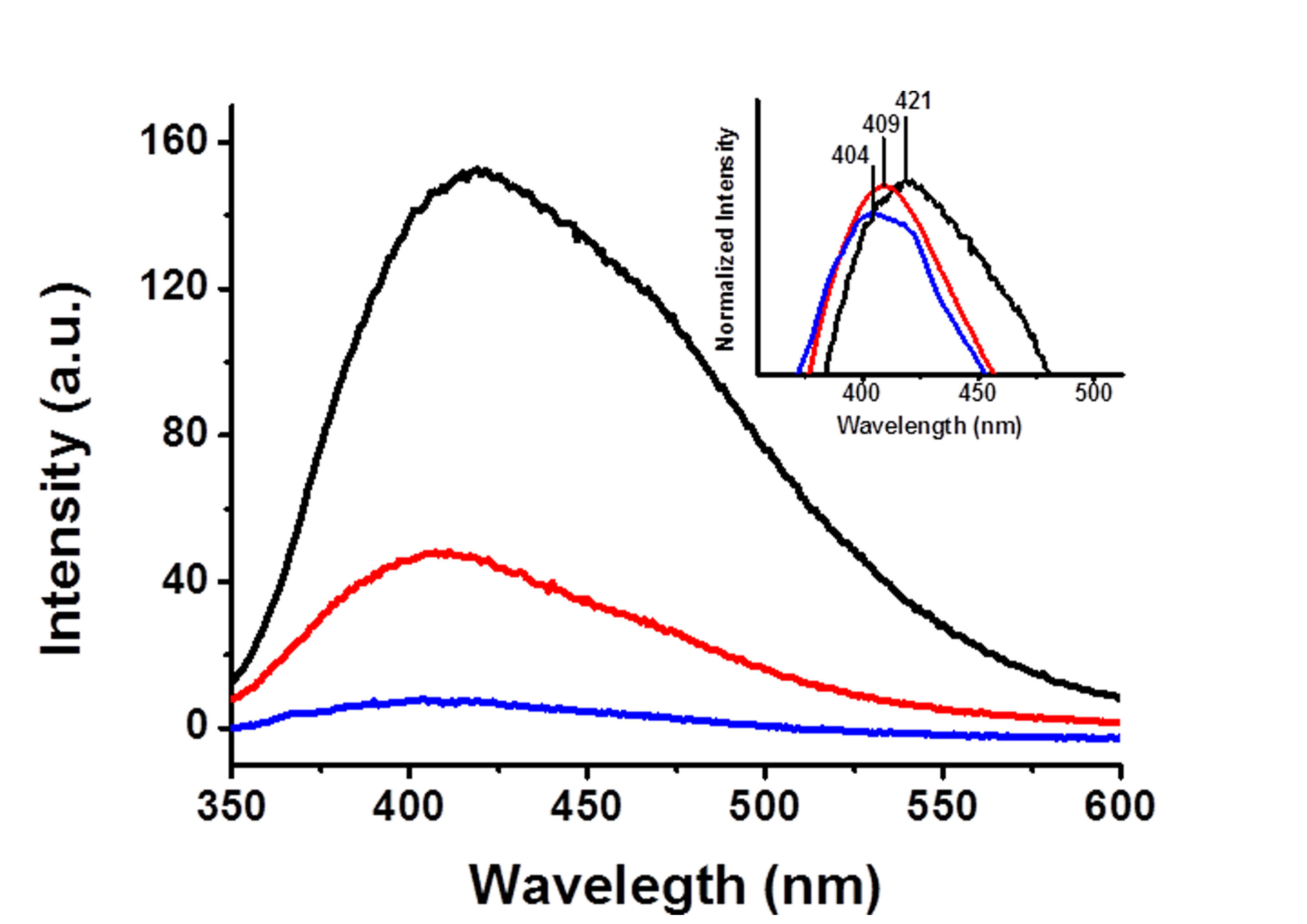


**Figure S4.** Photo-luminescence spectra of HFHPB-GQDs with increasing HFHPB diazonium salt content on a fixed amount of GQD using a 1mg/ml solution of HFHPB-GQDs: GQDs (black), HFHPB-GQDs (red)-HFHPB-N2+ : GQDs= 0.5 : 1, HFHPB-GQDs (blue)-HFHPB-N2+ : GQD= 5 : 1, ratios are by weight.

|  | Everage of lifetime τ_avg_ (ns) | τ_1_ (ns) | τ_2_ (ns) | τ_3_ (ns) |
| --- | --- | --- | --- | --- |
| GQD | 5.79 | 0.858 | 2.901 | 8.647 |
| HFHPB-GQD | 4.84 | 0.639 | 2.449 | 7.256 |
| HFHPB-GQD with DMMP | 5.05 | 0.526 | 2.308 | 7.304 |

**Table S1.** The fluorescence decay of GQD, HFHPB-GQD, and HFHPB-GQD interacted with DMMP (λ_ex_ = 340 nm, λ_em_ = 405 nm).


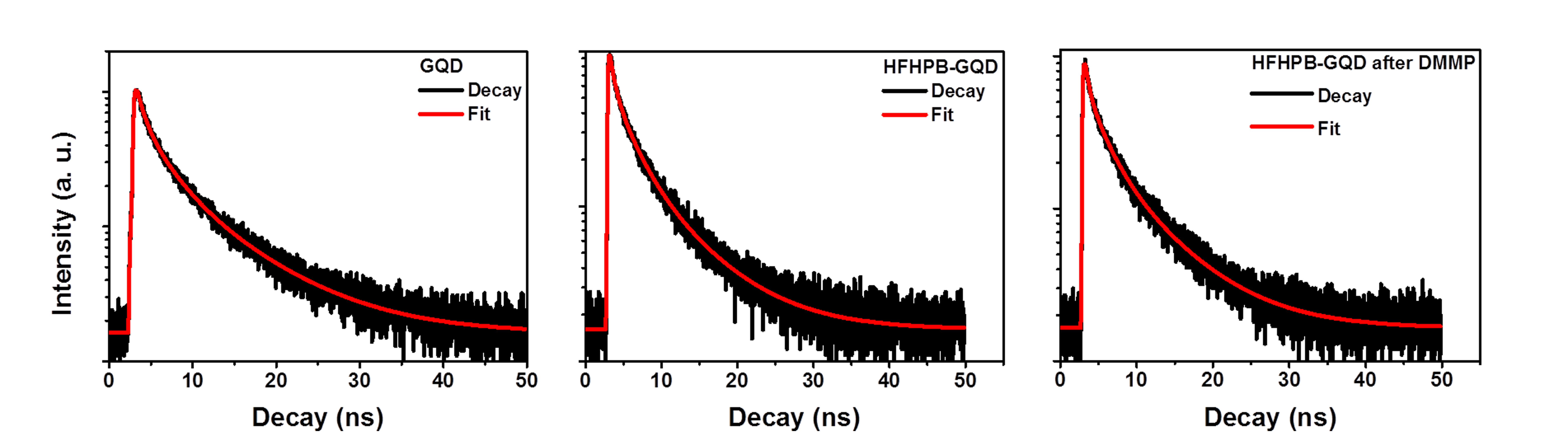


**Figure S5.** Lifetime of GQD, HFHPB-GQD, and HFHPB-GQD interacted with DMMP.

a.


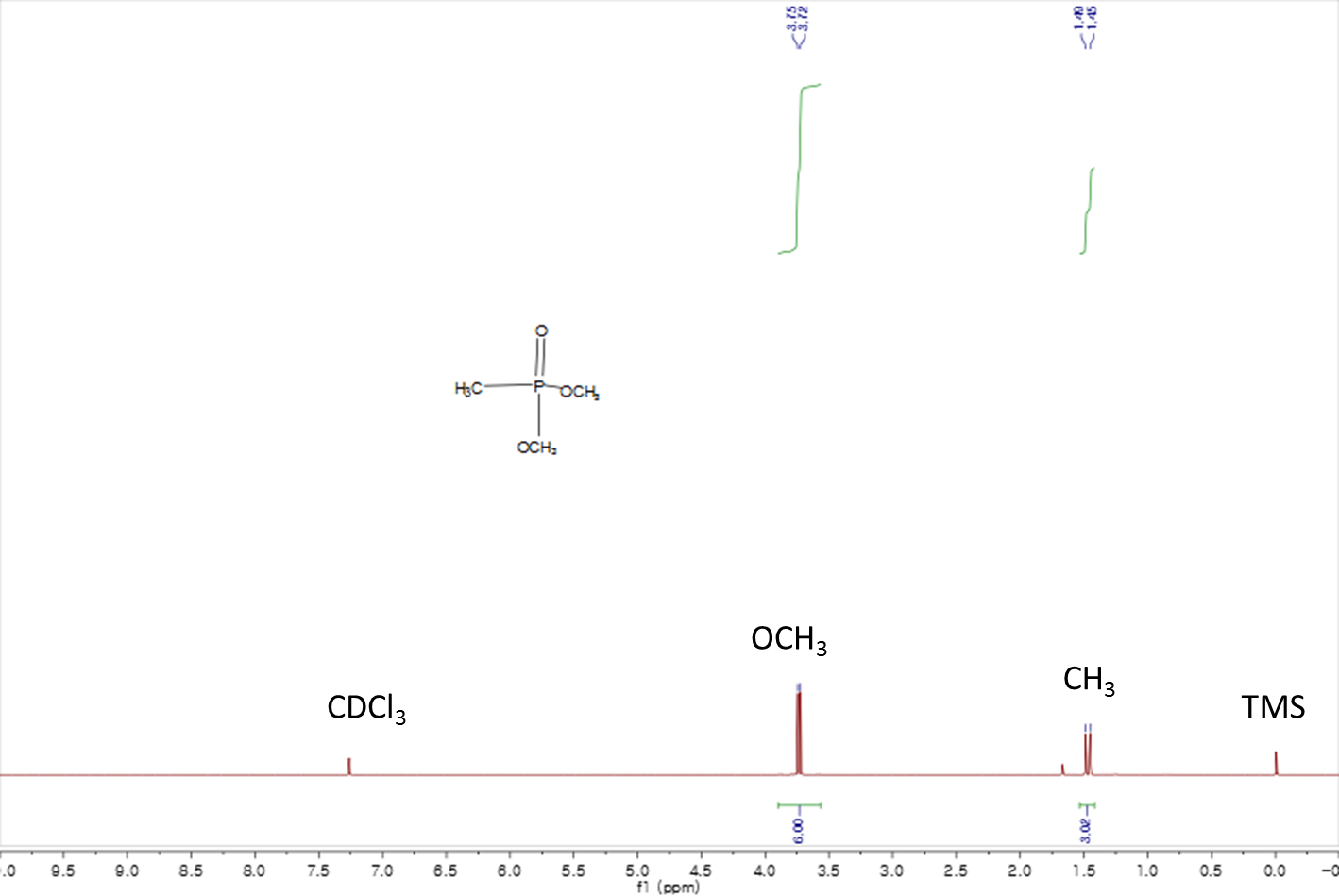


b.


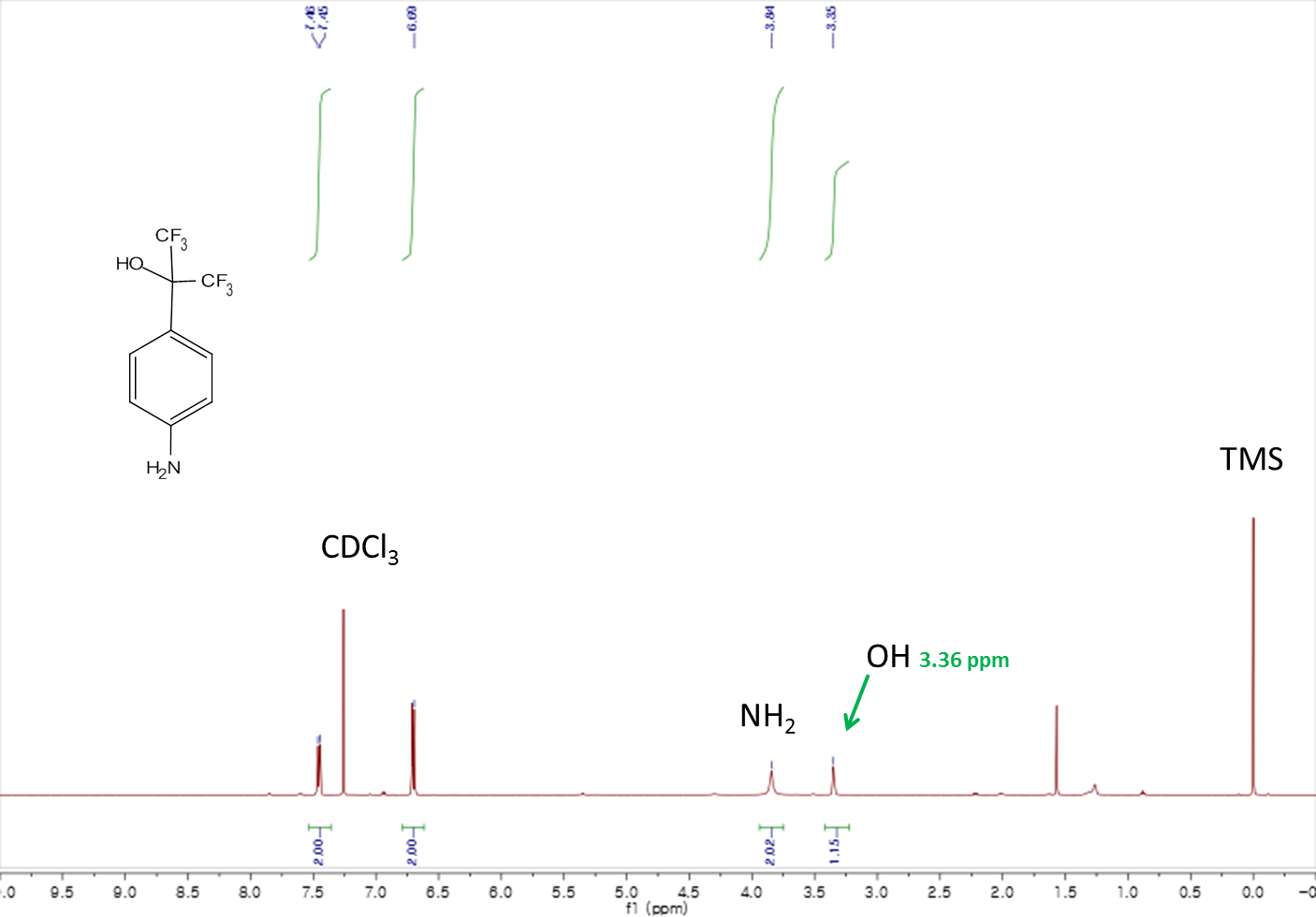


c.
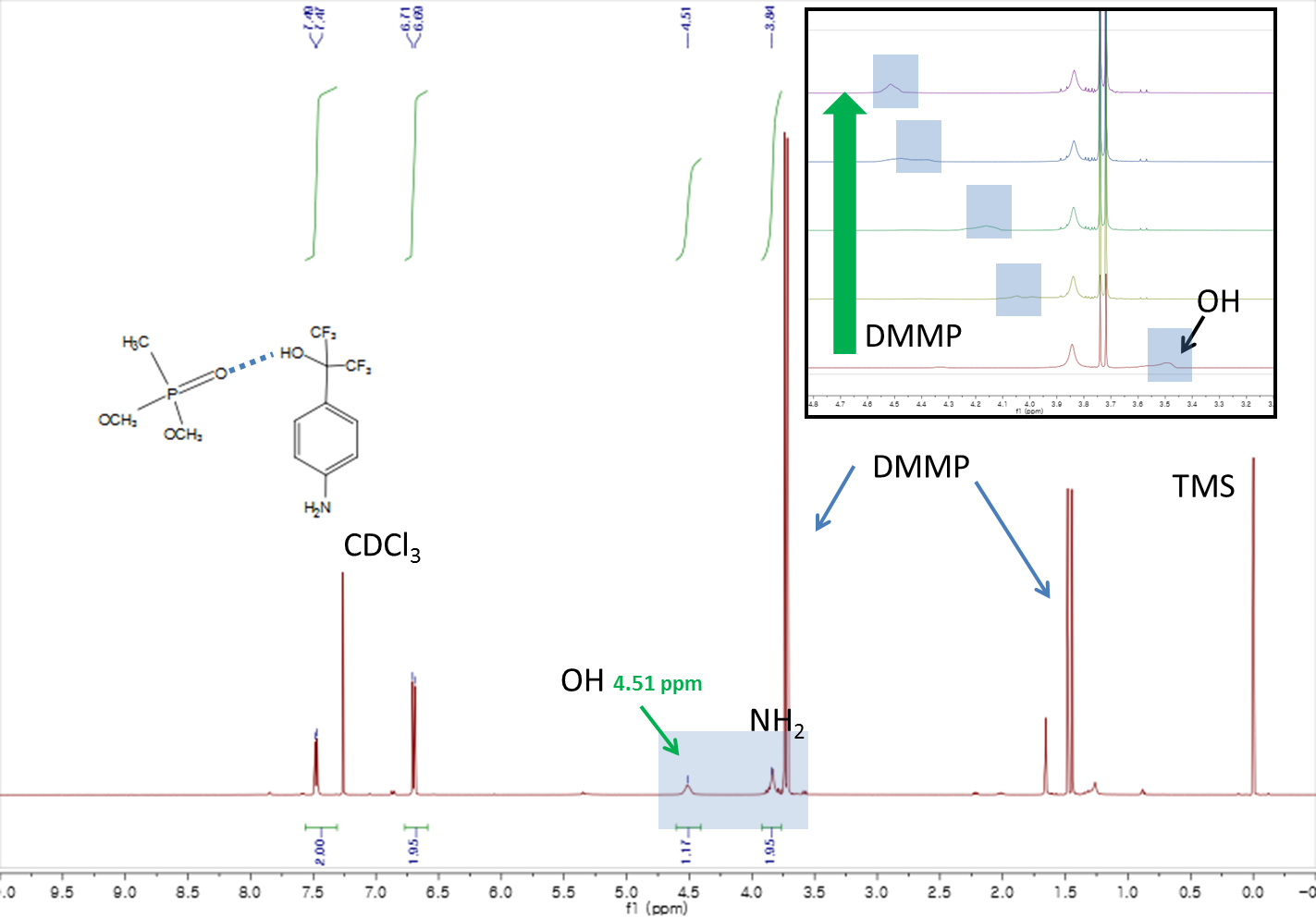


d.


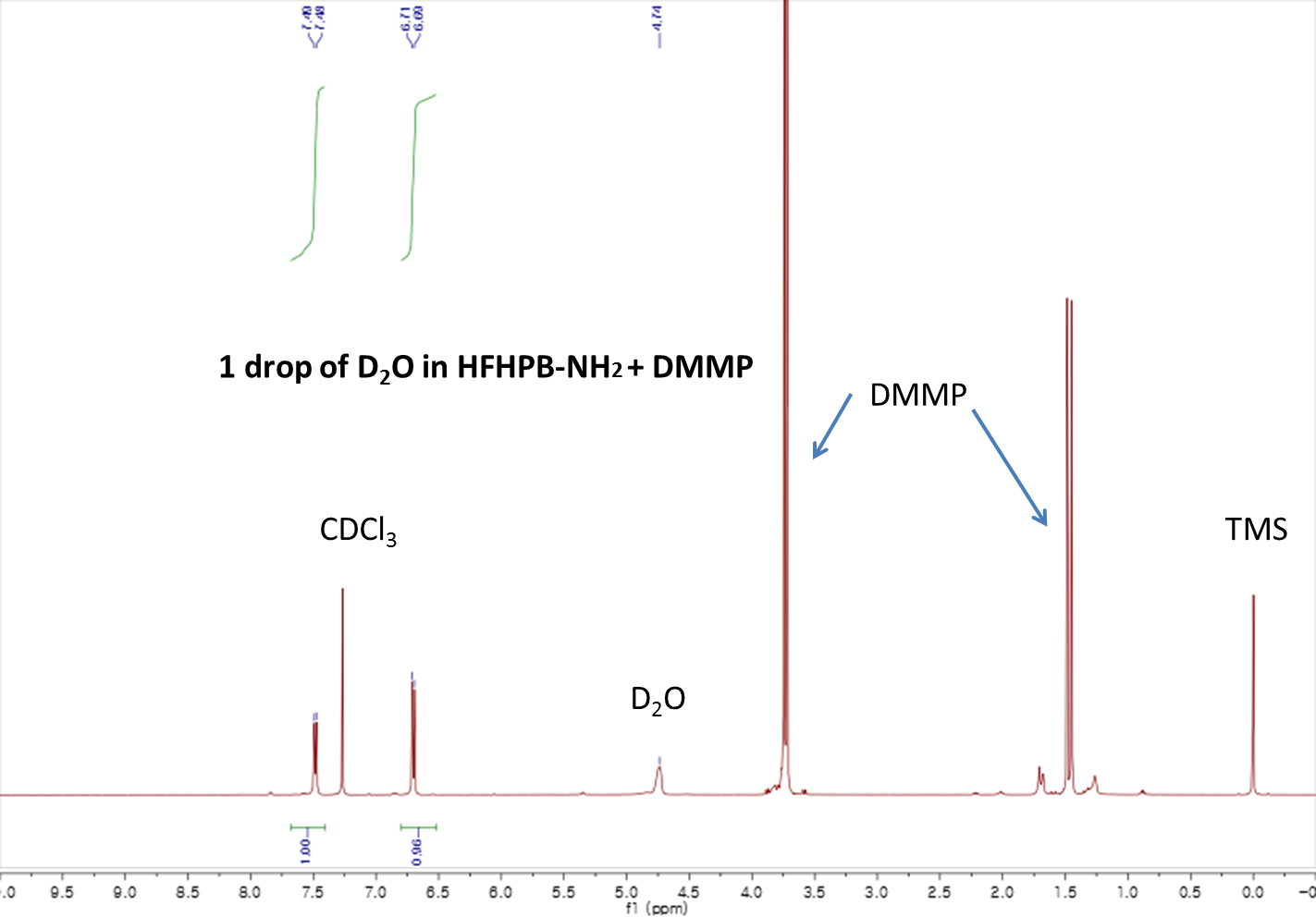


e.


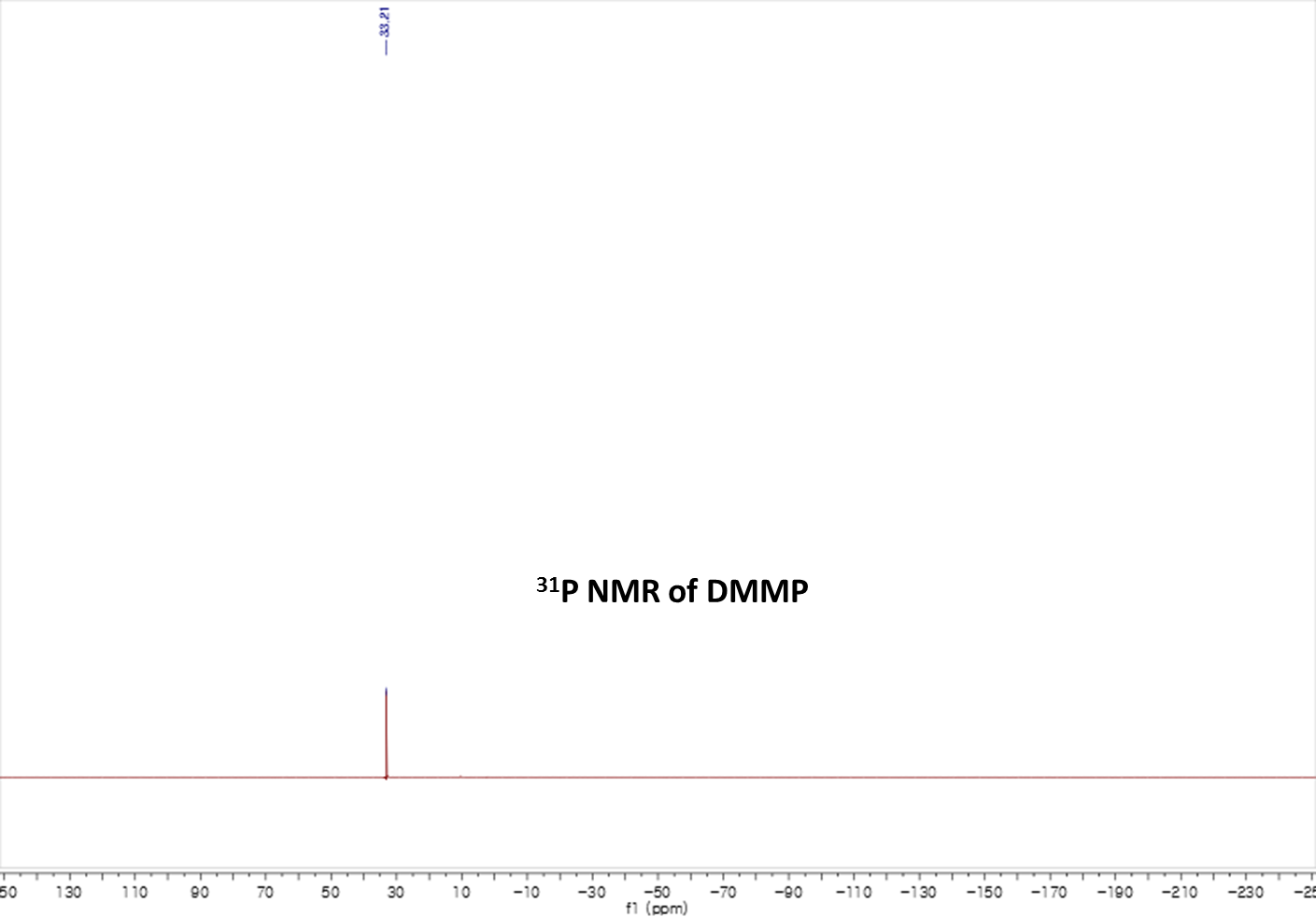


f.

**
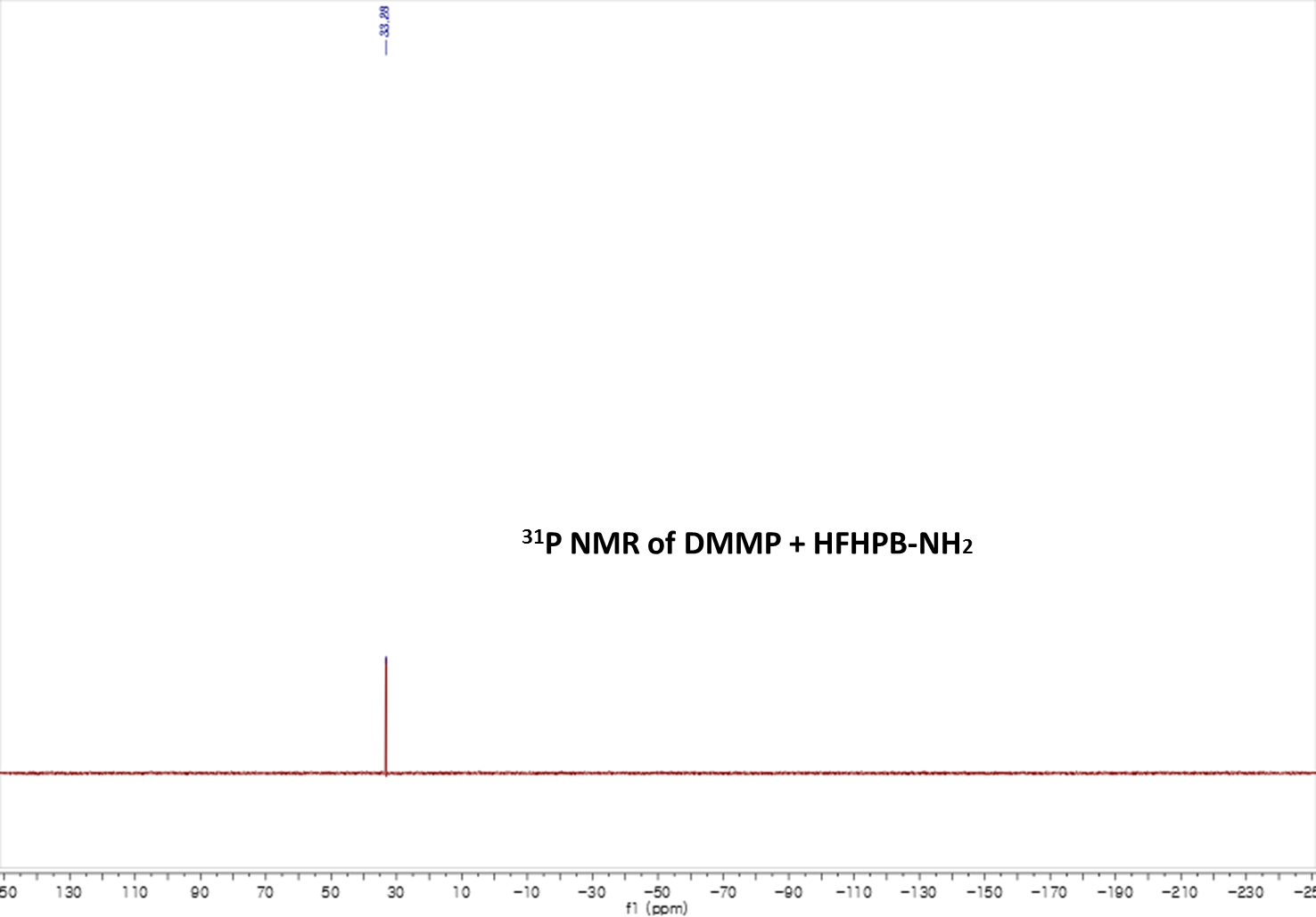
**

**Figure S6.** ^1^H NMR of DMMP (a), HFHPB-NH_2_ (b), HFHPB-NH_2_ with DMMP, inset : proton shift as increasing the mount of diluted DMMP (c), Deuterium exchange of HFHPB-NH_2_ and DMMP in D_2_O (d), ^31^P NMR spectrum of DMMP (e), and ^31^P NMR spectrum of HFHPB-NH_2_ and DMMP in CDCl_3_ (f).

**Characterization of detection of DMMP on HFHPB grafted GQDs by nanogravimetry**

The changes in frequency (Δƒ) were induced from a total change in mass (Δm) using the Sauerbrey equation^1)^, where the number of the harmonic (n) is 1, the resonant frequency (ƒ_0_ ) is 10 MHz, the active crystal area (A) is 0.28 cm^2^, the density of quartz (ρ_q_) is 2.648 g cm^-3^, and the shear modulus of quartz for the AT-cut crystal (μ_q_) is (2.947 x 10^11^ g cm^-1^ s^-2^), giving Δm = - 0.63 x 10^-9^ x Δƒ.


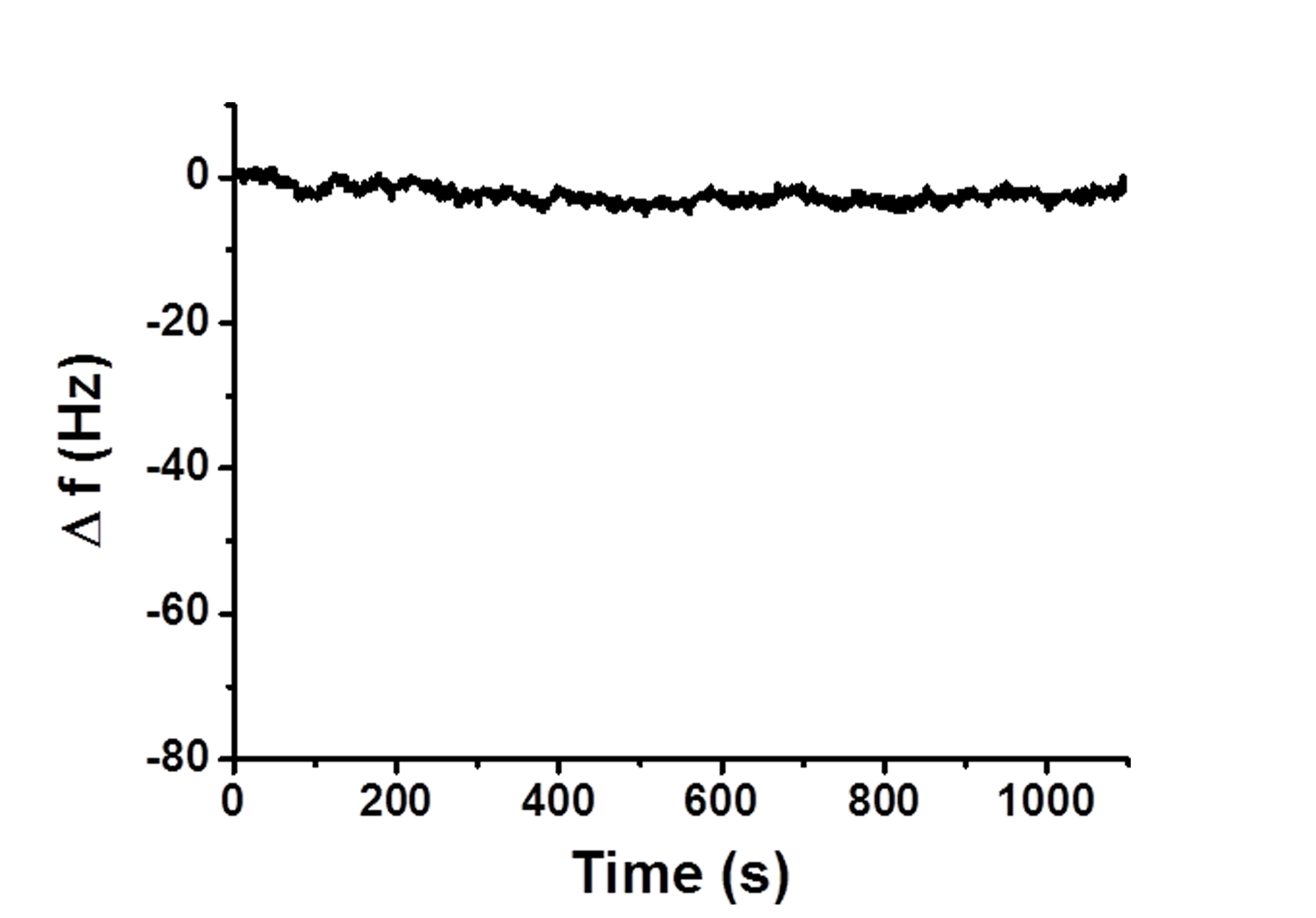


**Figure S7.** Nanogravimetry test with uncoated bare quartz crystal electrode.


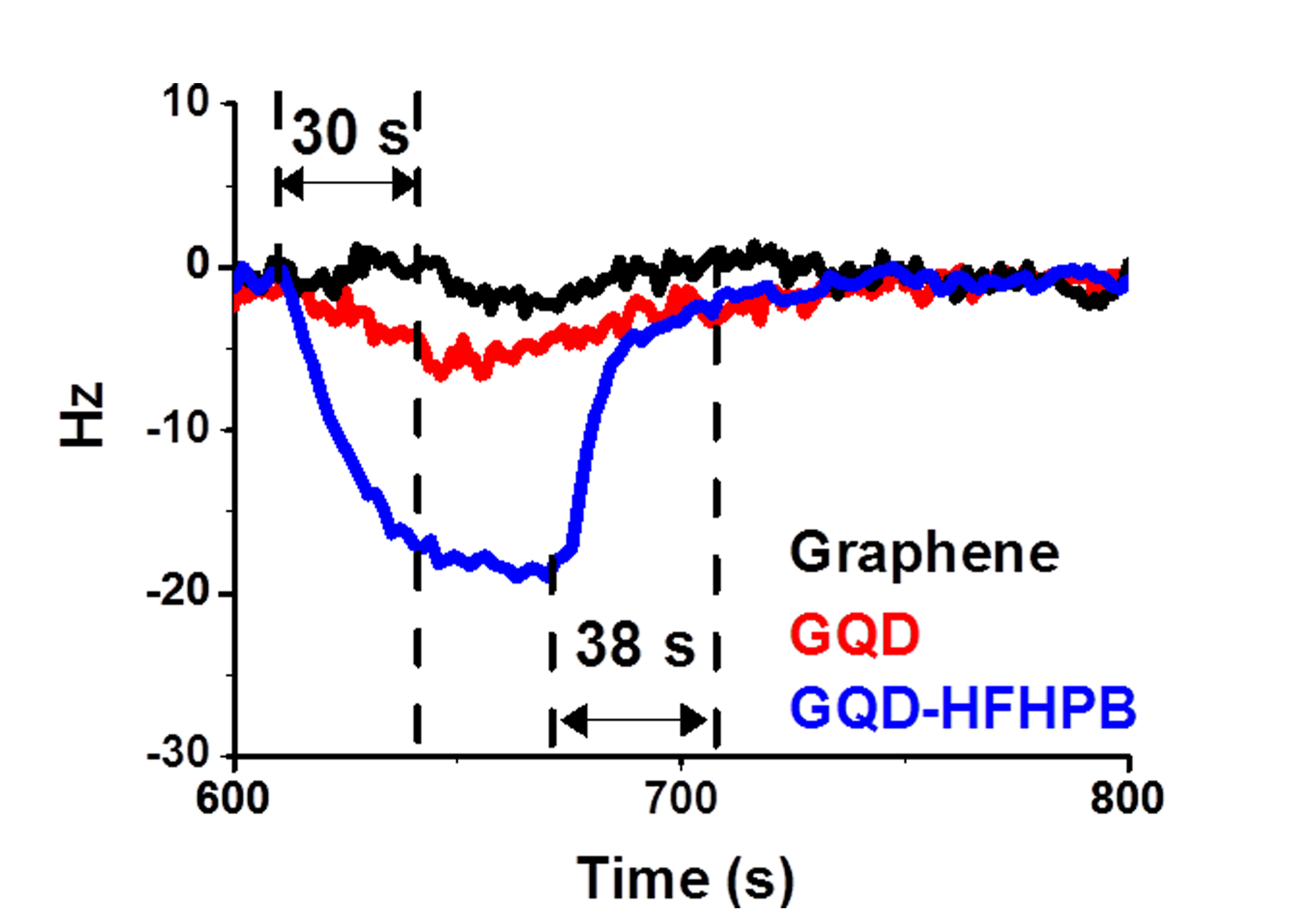


**Figure S8.** Magnified response and recovery time of HFHPB-GQDs at 8 ppm of DMMP.

|  | 90 % of absorption time | | 90 % of desorption time | |
| --- | --- | --- | --- | --- |
|  | HFHPB-GQD | GQD | HFHPB-GQD | GQD |
| 4 ppm | 31 | X | 36 | X |
| 8 ppm | 30 | 43 | 38 | 43 |
| 16 ppm | 32 | 55 | 36 | 33 |
| 32 ppm | 30 | 60 | 26 | 53 |
| Average of time | 31 | 53 | 34 | 43 |

**Table S2.** The response and recovery time on exposure of DMMP at 8 ppm.

1. S. Seo, M. Min, S. M. Lee and H. Lee, Photo-switchable molecular monolayer anchored between highly transparent and flexible graphene electrodes, Nat Commun., 4, 1920 ( 2013)
